# Complex Odontoma: A Case Report with Micro-Computed Tomography Findings

**DOI:** 10.1155/2016/3584751

**Published:** 2016-05-17

**Authors:** L. A. N. Santos, L. J. Lopes, G. D. Roque-Torres, V. F. Oliveira, D. Q. Freitas

**Affiliations:** ^1^Department of Oral Diagnosis, Division of Oral Radiology, State University of Montes Claros, Avenida Dr. Ruy Braga, N/N, Vila Mauriceia, 39401-089 Montes Claros, MG, Brazil; ^2^Piracicaba Dental School, State University of Campinas, Avenida Limeira 901, Areião, 13414-903 Piracicaba, SP, Brazil; ^3^Department of Maxillofacial Surgery, State University of Montes Claros, Avenida Dr. Ruy Braga, N/N, Vila Mauriceia, 39401-089 Montes Claros, MG, Brazil; ^4^Department of Oral Diagnosis, Division of Oral Radiology, State University of Campinas, Avenida Limeira 901, Areião, 13414-903 Piracicaba, SP, Brazil

## Abstract

Odontomas are the most common benign tumors of odontogenic origin. They are normally diagnosed on routine radiographs, due to the absence of symptoms. Histopathologic evaluation confirms the diagnosis especially in cases of complex odontoma, which may be confused during radiographic examination with an osteoma or other highly calcified bone lesions. The micro-CT is a new technology that enables three-dimensional analysis with better spatial resolution compared with cone beam computed tomography. Another great advantage of this technology is that the sample does not need special preparation or destruction in the sectioned area as in histopathologic evaluation. An odontoma with CBCT and microtomography images is presented in a 26-year-old man. It was first observed on panoramic radiographs and then by CBCT. The lesion and the impacted third molar were surgically excised using a modified Neumann approach. After removal, it was evaluated by histopathology and microtomography to confirm the diagnostic hypothesis. According to the results, micro-CT enabled the assessment of the sample similar to histopathology, without destruction of the sample. With further development, micro-CT could be a powerful diagnostic tool in future research.

## 1. Introduction

The term odontoma was first described by Broca [1] as a benign odontogenic tumor of epithelial and mesenchymal origin characterized by slow growth and dental contents (enamel, dentin, cementum, and pulp) [2]. It is considered a tumor-like malformation (hamartoma), not a true neoplasm, in which all of the dental tissues are represented [3, 4]. Although odontomas are usually asymptomatic, there are some clinical indicators such as retention of deciduous teeth, no eruption of permanent teeth, expansion of cortical bone, and displacement of teeth. Other symptoms include numbness in the lower lip and swelling in the affected area [5].

Odontomas are usually small in size [3, 4, 6, 7]. The age at diagnosis is commonly in the second decade of life without gender predilection, occurring more often in the posterior region of the mandible [8]. Based on radiographic and microscopic characteristics, odontomas are subdivided into compound and complex types. The compound type is characterized by tooth-like structures arranged in an orderly fashion and the complex type is characterized by dental tissues in a disorderly pattern without any anatomic resemblance to a tooth [3, 9–14]. Odontomas are the most common benign tumors of odontogenic origin. They are normally diagnosed on routine radiographs, mainly due to the absence of symptoms [7]. Histopathologic evaluation confirms the diagnosis especially [15] in cases of complex odontoma, which may be confused with an osteoma or another highly calcified bone lesion on radiographs [6, 7].

The diagnosis could be established by radiography (panoramic radiography and/or intraoral radiographs) but cone beam computed tomography (CBCT) has played an important role in the diagnosis and identification of lesions [16, 17], thereby helping in treatment planning [8].

Modern high-resolution micro-computed tomography (micro-CT) is increasingly used in dental research. This new technology enables three-dimensional analysis with better spatial resolution compared with CBCT. In addition, a great advantage of micro-CT is that the sample needs no special preparation; the only demand is that it is small enough to be mounted in the scanner [18]. A wide range of specimens may be examined directly including mineralized tissues, such as teeth and bone and materials such as ceramics, polymers, and biomaterial scaffolds [19, 20]. The morphological characteristics provided by micro-CT include the assessment of the mineral concentration of teeth with high accuracy (coefficient of variation < 1%), resolution of 5–30 *μ*m [21–23], and assessment of mineral density, all without sample destruction unlike the histopathological exam.

This paper presents a case report of a complex odontoma focusing on the micro-CT findings and highlighting the advantages of this new technology.

## 2. Case Report

A 26-year-old man was referred to the Department of Maxillofacial and Oral Surgery, Montes Claros University, with the chief complaint of pain in the region of the posterior right maxilla. Clinical examination revealed a swelling in the region of the upper right third molar shrouded by a large yellowish-brown hard mass, which resembled dentin, and a secretion-draining tract.

**Figure 1:**
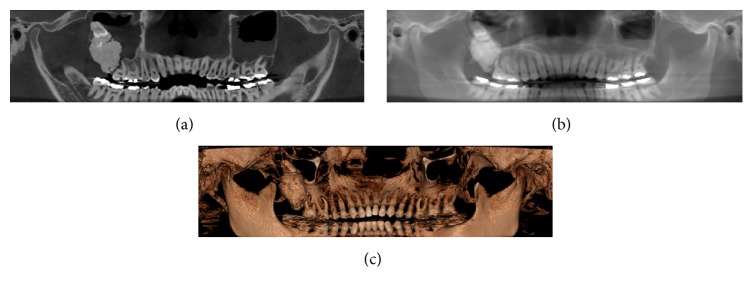
Panoramic radiograph reconstructed of CBCT. (a) With a thickness of 1 mm. (b) With a thickness of 10 mm. (c) 3D panoramic reconstructed.

Panoramic radiographs reconstructed of CBCT showed an irregular radiopaque mass surrounded by a distinct radiolucent rim associated with the upper right third molar, which was dislocated into the maxillary sinus (Figure 1). The first diagnostic hypothesis was a complex odontoma, but osteoma and other calcified bone lesions were not ruled.

**Figure 2:**
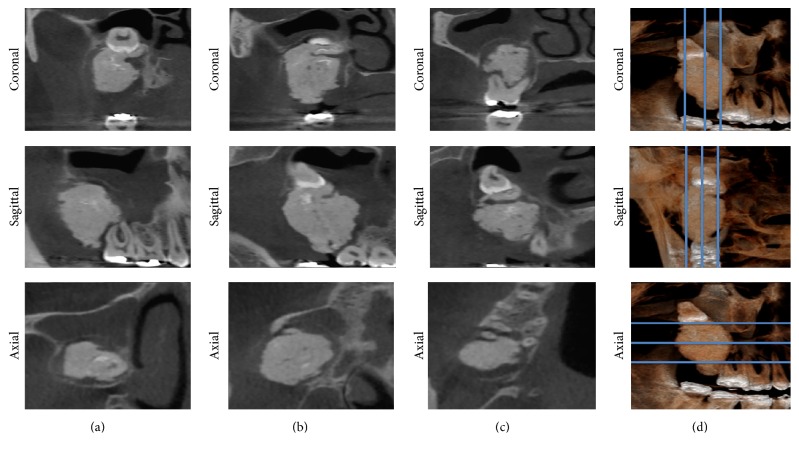
Multiplanar reconstruction of CBCT in three different regions from each view.

In anticipation of surgical excision of the lesion, CBCT images were obtained. The CBCT images revealed a 3.2 × 2.3 × 2.2 cm hyperdense mass interspersed with areas of hypodensity. The lesion extended superiorly from the alveolar ridge to the middle third of the right maxillary sinus, displacing the upper right third molar to the top. Laterally, the lesion extended toward the infratemporal fossa and a slight displacement of the medial wall of the right maxillary sinus was noted. Hyperdense areas resembling teeth were observed in the lesion. Thickening of the soft tissues with obstruction of the drainage pathways of the maxillary sinus was also observed. Coronal images demonstrated the extent of the internal epithelium of the maxillary sinus swelling suggesting sinusitis. The presence of fluid levels was seen in the left maxillary sinus, diagnostic of a sinus pathology (mucosa thickening) (Figure 2). The diagnostic hypothesis of a complex odontoma remained.

**Figure 3:**
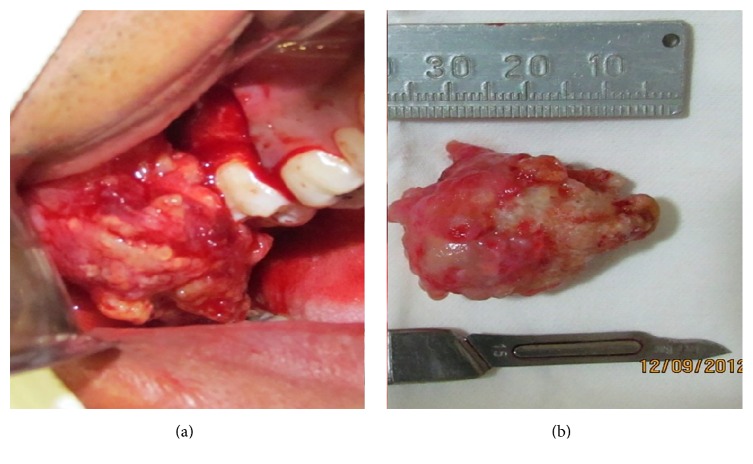
(a) Odontoma removal. (b) Complex odontoma.

Under local anesthesia, the lesion and the impacted third molar were surgically excised using a modified Neumann approach (Figure 3).

**Figure 4:**
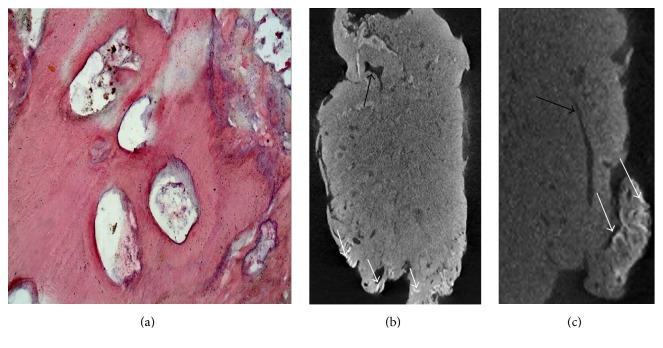
(a) Histopathology of the odontoma showing dense acellular eosinophilic dentin; basophilic dentin; prominent tubular array and minimal loose mesenchyme (hematoxylin and eosin stain). (b-c) Micro-CT images of the complex odontoma. White arrows, hyperdense areas; black arrows, hypodense areas. (b) Without zoom. (c) With zoom.

The specimen was sent for histopathological and micro-CT evaluation. Histologic sections revealed a mixture of radiopaque material composed mainly of dental tissues, consisting of immature dentin, enamel, enamel matrix, cementum, and pulp tissue. Histopathologic examination confirmed the diagnosis of complex odontoma (Figure 4(a)) [24].

A micro-CT scan of the sample (approximately 30 mm × 47 mm) was obtained using a SkyScan 1172 machine (Bruker SkyScan, Aartselaar, Belgium). A greater amount of hyperdense material in the periphery of the lesion was observed (Figures 4(b) and 4(c), white arrows), which is compatible with tooth enamel. Hypodense areas (Figures 4(b) and 4(c), black arrows) are compatible with areas of pulp tissue. The definitive diagnosis was complex odontoma.

In addition to the visual evaluation of the micro-CT images, we evaluated the density, volume, and surface. For this purpose, the sample was scanned at 50 kV, 800 *μ*A beam intensity, 30 *μ*m image pixel size, a 0.4° rotation step, 3-frame averaging, and a 411 ms exposure time at each step. A 0.5-mm aluminum filter was used during the scans. The images were reconstructed with NRecon (Bruker SkyScan, Aartselaar, Belgium). Polynomial correction was used to reduce smoothing, ring, and beam-hardening effects during the reconstructions [25–27].

The odontoma was assessed qualitatively with CTAn software (SkyScan, v. 1.4). The volume of interest (VOI) was designed by drawing polygons interactively on the two-dimensional gray images. Polygons were drawn in five sections (i.e., all the sample), and a routine utility calculated all the intermediary masks by interpolation. The VOI comprised only odontoma tissue and the following parameters were evaluated: tissue volume, bone volume, tissue surface, bone surface, bone surface density (hard tissue surface/tissue volume), total porosity, total volume of pore space, mineral concentration, density of the whole sample, and the value gray scale.

In the present case, the evaluation of the sample by micro-CT showed the parameters and values in Table 1.

## 3. Discussion

Odontomas are common odontogenic tumors and are usually asymptomatic [17]; however, in this case, the patient experienced pain in the region of the third molar, probably due to the presence of inflammatory component of the lesion with exudate drainage. Odontomas rarely erupt [24] but when this occurs, they are different from a normal tooth because of the lack of the periodontal ligament. The increase in size leads to sequestration of the overlying bone, causing pressure and possible movement in the occlusal direction, leading to eruption.

In this case, the tumor led to retention of the right third molar, which is a common characteristic in odontomas, although not reported in clinical cases with tuberosity of the maxilla [12], because there is high predilection of complex odontoma in the posterior region of the mandible [12, 24]. Most complex odontomas reported in the literature typically measure around 1-2 cm [3]. A large complex odontoma as in this case (3 × 4.7 cm) is rare [4, 7, 14].

The diagnosis of compound odontoma can usually be established by conventional radiographic examination (intraoral or panoramic radiographs) performed for another reason when the lesion is small [17]. However, this is not usually the case with complex odontomas, because they may be confused with other tumors, such as cementoid tumors and several other bone lesions [8]. For that reason, CBCT was performed, which also aided surgical planning. The scan was very useful to visualize the precise relationship between the lesion and the third molar [5, 17]. As demonstrated by other studies, early diagnosis of odontomas allows for less complex treatment, guaranteeing a better prognosis [17].

As a relatively new method of high-resolution three-dimensional imaging, micro-CT has been widely used in many academic fields. Many studies have presented the current state of micro-CT imaging [18, 27–30]. The images represent spatial distribution maps of linear attenuation coefficients determined by the energy of the X-ray source and the atomic composition of the material sample, without preparation or destruction of the sample in the area to be sectioned [27]. Micro-CT has become more and more used for the analysis of mineral concentrations of teeth. Accurate and detailed images of the morphological characteristics of teeth are provided by micro-CT scans in dental research; the mineral concentration of teeth can be measured with high accuracy, images at a resolution of 5–30 *μ*m are obtained, and mineral density can be indirectly assessed [23].

In this case, the sample had 957 slices, the total tissue volume was 6506.8 mm^3^ (100%), and the hard tissue volume was 5719.3 mm^3^ (87.9%). The soft tissue surface was 3193.1 mm^2^ and the hard tissue surface was 9758.8 mm^2^. The density of the sample was 0.92 g/cm^3^ (0.27 g/cm^3^); this density is close to the density of circumpulpal dentin (1.3 g/cm^3^) and the mineral concentration of the sample was 0.69 g/cm^3^, similar to demineralized dentin (0.55 g/cm^3^). The gray scale value of the sample was found 93.38 (15.80), similar to that of rigid dentin [31].

Histopathologically, complex odontomas are composed of a mixture of dental tissues, mainly forming a single homogeneous mass of immature dentin, enamel, enamel matrix, cement, and pulp tissue in a random fashion. The micro-CT images of this case showed results consistent with histopathological evaluation, with a large volume of hard tissue and a mineral density similar to human immature dentin [31, 32]. This case report also demonstrates the possibility of clinical application of this imaging modality with good visualization. However, more studies are necessary to elucidate whether micro-CT analysis is valuable in the management of the odontoma.

## 4. Conclusions

This case report demonstrates the use of advanced diagnostic imaging, such as CBCT, which allowed a more appropriate treatment plan for excision of the lesion. In addition, micro-CT enabled the assessment of the sample similar to histopathology, without destruction of the sample. With further development, micro-CT could be a powerful diagnostic tool in future research.

## Figures and Tables

**Table 1:** Parameters and values of the micro-CT findings.

Parameters	Values
Tissue volume (TV)	6506.8 mm^3^
Bone volume (BV)	5719.3 mm^3^
Percent bone volume (BV/TV)	87.9%
Tissue surface (TS)	3193.1 mm^2^
Bone surface (BS)	9758.8 mm^2^
Bone surface density (BS/TV)	1.49979 1/mm
Total porosity (percent) (Po(tot))	12.1%
Total volume of pore space (Po.V(tot))	787.593.38 mm^3^
Mineral concentration	0.69 g/cm^3^
Density	0.92 (0.27)
Gray scale	93.38 (15.80)
